# P18 Steroid-induced malignant hypertension causing cytopenia in a case of lupus nephritis

**DOI:** 10.1093/rap/rkab068.017

**Published:** 2021-10-19

**Authors:** Deena Laila, Ziad Alkutobi, Anurag Bharadwaj

**Affiliations:** Basildon University Hospital Mid &South Essex NHS Trust Group, Basildon, United Kingdom

## Abstract

**Case report - Introduction:**

Proteinuria, hypertension and renal impairment are among the features of lupus nephritis, whereas seizure and cytopenia are part of systemic manifestations of active lupus. In these situations, high-dose steroid is usually considered and can lead to severe/malignant hypertension (MHT). We are reporting a case of lupus nephritis admitted with seizures and later developed thrombotic microangiopathy (TMA). Further evaluation revealed a diagnosis of steroid-induced malignant hypertension which was later improved after controlling blood pressure and reducing the dose of steroid.

**Case report - Case description:**

A 25-year-old lady who was previously diagnosed at 17 years old with systemic lupus erythematosus (SLE) based on clinical features of polyarthralgia, lymphadenopathy, fatigability and positive auto immune profile. She was lost to follow up since the diagnosis and had not been on relevant treatment.

She was recently admitted with myalgia, polyarthralgia, pleural effusion and leg oedema. Blood test showed stage 2, acute kidney injury and urine analysis showed significant proteinuria (PCR 826mg/mmol). Renal biopsy showed class IV lupus nephritis and she was commenced on prednisolone 50 mg orally and was discharged home with outpatient follow up appointment.

A few days later, she presented with generalized tonic clonic seizure. Physical examination revealed high blood pressure at 160/120 mmHg. MRI brain scan did not show features of ischaemia or thrombosis. She was treated with antiepileptic, antihypertensive medications and parenteral methylprednisolone 500 mg in view of lupus with neurological manifestation. While being on methylprednisolone, her blood pressure further rose with systolic 220/ diastolic 170 mmHg. Her blood parameter started deteriorating showing features of microangiopathic haemolytic anaemia (MAHA) with low haemoglobin of 56 and low platelets of 46. Blood test for anti-cardiolipin antibody, lupus anticoagulant were negative and marker for thrombotic thrombocytopenic purpura (TTP) Serum ADAMTS13 (A Disintegrin and Metalloprotease with a Thrombospondin type1 motif, member 13) was normal. Haematology team’s opinion was sought and TTP ruled out as a cause of her anaemia and thrombocytopenia. Repeat blood test did not show any features of worsening lupus and urine analysis showed improved proteinuria. Management plan was focused on controlling her blood pressure with optimization of antihypertensive medications. Parenteral steroid therapy was stopped. Within a few days of achieving good control of blood pressure, cytopenia improved. Antiepileptic treatment was stopped and she did not have any further episodes of seizure.

**Case report - Discussion:**

In assessing a case of lupus nephritis who appeared to have MAHA and thrombocytopenia with severe HTN, clinicians need to consider possibility of TMA along with other common differentials such as active lupus, TTP and haemolytic uraemic syndrome (HUS). In this case lupus nephritis and neurological manifestation (seizure) diverted attention towards active lupus. Patient was treated with parenteral steroids in order to control active lupus which caused worsening haematological parameters. Steroid-induced malignant hypertension can be difficult to consider as a differential diagnosis in these situations and can be easily missed.

TMA is a rare but serious condition which describes a specific pathological abnormality in the vessel wall of arterioles and capillaries leading to microvascular thrombosis. It is commonly manifested as MAHA and thrombocytopenia in blood film which indicates underlying endothelial injury. This case’s blood film showed thrombocytopenia and red blood cell fragmentation suggestive of MAHA. Malignant hypertension is one of the underlying causes of TMA where blood pressure control would be the most critical and may be the only management required to reverse the haematological abnormality as was evident in this case.

**Case report - Key learning points:**

Cytopenia in a case of SLE can be due to autoimmune process or drug-induced. However, malignant hypertension can also cause thrombotic microangiopathy which can lead to cytopenia. Thus, clinicians should be vigilant and relate this complication to the high dose of steroid.

